# Cook County Hospital

**Published:** 1877-10

**Authors:** R. Park

**Affiliations:** House Physician


### Clinical Reports.

### COOK COUNTY HOSPITAL.

### Service of Dr. H. A. Johnson.

[Reported by R. Park, House Physician.]

*A Case of Pericarditis Suppurativa.*

James Kennedy, Act 45. Admitted June 4th. Patient contracted syphilis twenty-two years ago, but no secondary or tertiary symptoms that he can recall followed. Aside from this he has always enjoyed good health, although addicted to the use of stimulants. Five days ago after a hard day's work he exposed himself to cold while perspiring freely. Soon after he had a chill, followed by fever, sleeplessness; nausea and loss of appetite. The next day he began to cough, and suffered from general malaise. The third day he expectorated a tenacious, white sputum; most of the other symptoms having in the meantime subsided. Since then the cough has been aggravating, expectoration more profuse, and the act of coughing has given considerable pain.

He is large, muscular, well nourished, face flushed, skin hot and dry, pulse 100, temp. 102°, resp. 30. The bowels are very costive. Physical examination gives the following notes: —chest well formed, expansive movements good, respiration hurried and jerking; percussion resonance good over the entire chest, vocal resonance normal; dry, coarse rales on both sides, best marked in right mammary region and vicinity. No friction sounds; heart sounds muffled and feeble, but normal so far as can be known.

He was given a general tonic and laxative treatment, with an anodyne expectorant.

On the 10th the following note was made: Patient's countenance has a peculiar, wild, staring appearance; tongue lightly coated, pulse 102, face and body bathed in profuse perspiration, bowels as before.

14th. Patient mutters and talks constantly during sleep, which is very much broken. Sweats profusely, circulation very weak. Stimulants were exhibited p. r. n. Without giving the daily notes *verbatim*, the following will show the progress of the case.

17th. Dulness, feeble vocal resonance and very feeble vesicular murmur over lower half of right chest; exaggerated respiration over opposite side.

18th. Above physical signs intensified; patient much prostrated and suffering from dyspnœa.

19th. Considerable bulging of affected side. Pain and dyspnœa aggravated. The usual treatment for alleviating such symptoms instituted.

20th, 3 A. M. Was called suddenly to see patient. Found him with fluttering pulse and suffering from excessive dyspnœa, face cyanosed, breathing short and hurried, anxious countenance, and bathed in cold, clammy perspiration.

Being first satisfied—by trial with the hypodermic syringe—that there was a collection of fluid on the affected side, I introduced the aspirator needle in the axilla in the sixth intercostal space, and withdrew f ℥ lvi. of sanguinolent serum, which coagulated as soon as exposed to the air. This relieved him very much for the time.

11, A. M. Patient slept for a few hours after the above oper ation, to awake at eight and rapidly sink. At that hour the respirations were 41, pulse fluttering and irregular, face cyanosed. Powerful stimulants were administered, but with no effect, and he expired at 10:45 A. M.

*Autopsy*: 24 hours after death. Face still livid; tissues well cushioned with fat. Upon opening the thorax both pleural cavities were found to contain a considerable quantity (O jss each?) of bloody serum; still no small part of the lung was adherent to the thoracic walls, while the right lung adhered to the diaphragm as well as to the thoracic walls,—in places. Further examination seemed to indicate that these adhesions were not of recent origin.

The lungs presented a few scattered patches of pneumonic consolidation, by no means regularly disposed. In the lower left lobe was found an incapsulated caseous mass which creaked under the knife. The pericardial sac with contents was found to present a mass much larger than normal. On nicking it with the knife pus welled out, and upon opening it further exit was given to about a pint of pus. The heart was adherent to it in many places, and many large organized masses were found. Upon removal of the mass and further dissection the pericardium was found adhering to nearly the entire left heart except at the base, where another pocket of pus was found, similar to the one mentioned above, which was much larger and surrounded the right heart.

A dilation of the aorta just at its origin was found, which would have held a hen's egg. The walls of the heart were much thickened, but the valves were in fair condition. The aorta gave evidence of incipient atheromatous change.

The liver bulged up into the right chest. It was large and, upon section, glistened from the amount of fat it contained. It was of a pale, yellowish-red color. The abdomen contained about a quart of serum. Spleen normal. Kidneys showed atrophy of the cortical substance and fatty degeneration, the pyramids standing out well.

*Aneurism of Ascending Aorta; Rupture of Left Auricle.*

Lizzie Walker (col.), Aet 45. Admitted May 16th. About a year ago patient began to experience a difficulty in breathing, especially at night. Had been employed for some time previously as a laundress, and had frequently "caught cold."

Since that time she has been annoyed—particularly during inclement weather or her menstrual epochs—with dyspnœa. Often at night she is unable to preserve the recumbent posture, being compelled to sit up in order to inflate the lungs. Apart from the above her general health has been good; she has seldom been unable to work.

On admission she is well nourished, of large frame, and apparently in excellent condition, except that she is troubled with great dyspnœa. Physical examination gives the following points: Expiration prolonged, with a wheezy, jerking inspiratory note, and dry or moist rales over entire chest, the dry predominating. Area of cardiac dullness much increased, the apex being an inch to the left of the nipple. There is an irregular rhythm and a combination of sounds—at the same time muffled and indistinct—which it is difficult to satisfactorily explain.

She was given tonics and the ext. grindeliæ robust.

From the date of her admission her condition seemed to improve; though she was always much oppressed for breath at night or in stormy weather. But on the evening of June 6th, while sitting up in bed, some little incident occurred to arouse her temper (none of the sweetest) and she sank suddenly back, and in five minutes was dead; having been insensible during this short interval,—with tongue protruding and pulse fluttering at first but finally quietly subsiding.

*Autopsy;* 15 hours after death. Dura mater adherent to calvarium; membranes engorged. Considerable serum beneath pia mater. On opening the membranes and sinuses uncoagulated blood flowed freely; and after cutting the cord and removing the brain it continued to flow in such quantity as to show intense engorgement of the meningeal vessels of both brain and cord. Careful examination and section failed to reveal any rupture or hæmorrhage; but there was found a marked condition of atheromatous change in all the arteries so far as traced.

Upon opening the thorax, the pericardium was found distorted and enormously distended by fluid; section proved this to be semi-solid, half coagulated blood. The lungs and heart were then removed together; the former were engorged with blood, but no evidences of inflammatory action were to be seen. Careful dissection of the pericardial contents showed a marked roughening and thickening of the serous pericardial surfaces, not of recent origin. The whole mass, heart, clots and all, weighed about ℥lv. After turning out the blood and clots, a rupture of the left auricle was found. Following up this lead an aneurism of the commencement of the aorta was revealed, which also involved the integrity of the heart wall and inter-auricular septum. The right heart was very much hypertrophied, the left very much dilated. The cavity of the aneurism must have been about the size of a black-walnut. Patches of atheroma, with occasional calcareous patches, were seen all along the course of the thoracic aorta.

The abdominal viscera were normal.

In the uterus—which was of normal size—were found several small fibroid tumors, the largest of which was not more than 2 cm. in diameter. A similar but much smaller growth involved one extremity of the right ovary.

